# Supplementary material for: Sheep’s Second Cheese Whey Edible Coatings with Oregano and Clary Sage Essential Oils Used as Sustainable Packaging Material in Cheese
Source: Foods. 2024 Feb 23;13(5):674. doi: 10.3390/foods13050674 (PMC10931019; doi:10.3390/foods13050674)
Supplement: Supplementary file 1 [file foods-13-00674-s001.zip › foods-2851517-supplementary.pdf]

foods MDPI

Supplementary Materials

Table S1. Physicochemical parameters of cheese samples. Average values and standard deviation (±).

			**DRY MATTER (%w/w)**		
Storage time (days)	C	N	WC	WCO	WCS
1	52.9 ± 0.7 ^{a,A}	53.1 ± 0.6 ^{a,A}	53.8 ± 0.8 ^{a,A}	56.5 ± 1.0 ^{a,B}	54.3 ± 0.7 ^{a,A}
7	62.1 ± 2.5 ^{b,A}	64.7 ± 1.1 ^{b,A}	64.4 ± 4.7 ^{b,c,A}	65.3 ± 2.1 ^{a,A}	61.5 ± 3.7 ^{b,A}
14	61.8 ±3.2 ^{b,A}	63.7 ±1.6 ^{b,A}	61.1 ±3.2 ^{a,b,A}	63.5 ±1.1 ^{a,A}	62.7 ±1.1 ^{b,A}
21	63.4 ± 3.1 ^{b,A}	68.4 ± 4.1 ^{b,A}	71.6 ± 3.6 ^{c,A}	65.0 ± 5.6 ^{a,A}	66.6 ± 2.2 ^{b,A}
28	64.9 ± 4.5 ^{b,A}	65.6 ± 1.9 ^{b,A}	70.1 ± 4.1 ^{b,c,A}	60.5 ± 4.8 ^{a,A}	61.8 ± 1.3 ^{b,A}
			WATER ACTIVITY		
1	0.933 ± 0.0 ^{b,A,B}	0.932 ± 0.0 ^{ab,A}	0.944 ± 0.0 ^{c,C}	0.942 ± 0.0 ^{c,B,C}	0.938 ± 0.0 ^{b,A,B,C}
7	0.934 ± 0.0 ^{b,A,B}	0.939 ± 0.0 ^{b,B}	0.935 ± 0.0 ^{b,A,B}	0.936 ± 0.0 ^{b,A,B}	0.929 ± 0.3 ^{a,A}
14	0.937 ± 0.0 ^{b,A}	0.935 ± 0.0 ^{b,A}	0.936 ± 0.0 ^{b,A}	0.939 ± 0.0 ^{b,c,A}	0.936 ± 0.0 ^{ab,A}
21	0.933 ± 0.0 ^{b,A}	0.937 ± 0.0 ^{b,A}	0.933 ± 0.0 ^{b,A}	0.936 ± 0.0 ^{b,A}	0.937 ± 0.0 ^{ab,A}
28	0.921 ± 0.0 ^{a,A}	0.924 ± 0.0 ^{a,A}	0.920 ± 0.0 ^{a,A}	0.930 ± 0.0 ^{a,B}	0.932 ± 0.0 ^{ab,B}
			pH		
1	5.6 ± 0.1 ^{a,A}	5.5 ± 0.1 ^{a,A}	5.5 ± 0.1 ^{a,b,A}	5.5 ± 0.0 ^{a,A}	5.4 ± 0.1 ^{a,A}
7	5.6 ± 0.1 ^{a,A}	5.6 ± 0.1 ^{a,A}	5.6 ± 0.1 ^{a,b,A}	5.5 ± 0.1 ^{a,A}	5.5 ± 0.0 ^{a,b,A}
14	5.5 ± 0.1 ^{a,A}	5.6 ± 0.1 ^{a,A}	5.5 ± 0.0 ^{a,A}	5.5 ± 0.1 ^{a,A}	5.6 ± 0.1 ^{b,c,A}
21	5.6 ± 0.1 ^{a,A}	5.5 ± 0.1 ^{a,A}	5.7 ± 0.0 ^{c,B}	5.5 ± 0.1 ^{a,A}	5.5 ± 0.0 ^{a,b,A}
28	5.7 ± 0.1 ^{a,A}	5.5 ± 0.2 ^{a,A}	5.6 ± 0.1 ^{b,c,A}	5.4 ± 0.1 ^{a,A}	5.6 ± 0.0 ^{c,A}
			TITRATABLE ACIDITY (% lactic acid)		
1	0.37 ± 0.0 ^{c,C}	0.19 ± 0.0 ^{a,A,B}	0.21 ± 0.0 ^{a,b,A,B}	0.23 ± 0.0 ^{b,B}	0.14 ± 0.0 ^{a,A}
7	0.27 ± 0.0 ^{b,c,B}	0.22 ± 0.0 ^{a,A,B}	0.24± 0.0 ^{b,B}	0.24 ± 0.0 ^{b,B}	0.16 ± 0.0 ^{a,A}
14	0.22 ± 0.0 ^{a,b,C}	0.19 ± 0.0 ^{a,B,C}	0.13 ± 0.0 ^{a,A,B}	0.12 ± 0.0 ^{a,A}	0.12 ± 0.0 ^{a,A,B}
21	0.16 ± 0.0 ^{a,A}	0.17 ± 0.1 ^{a,A}	0.19 ± 0.1 ^{a,b,A}	0.12 ± 0.0 ^{a,A}	0.12 ± 0.1 ^{a,A}
28	0.21 ± 0.1 ^{a,b,B}	0.17 ± 0.0 ^{a,A,B}	0.13 ± 0.0 ^{a,A,B}	0.11± 0.0 ^{a,A}	0.13 ± 0.0 ^{a,A,B}

C-Control; N-cheese with natamycin; WC-cheese with SCW coating; WCO-cheese with SCW coating with oregano essential oil; WCS-cheese with SCW coating with clary sage essential oil. Different superscript letters (a,b,c) indicate significant differences for the same product in different ripening days. Superscript capital letters (A,B,C) indicate significant differences between products at the same ripening day.

Table S2. CIEL*a*b* parameters of cheese samples. Average values and standard deviation (±).

			**L*RIND**		
Storage time (days)	C	N	WC	WCO	WCS
1	92.0 ± 0.2 ^{d,A}	91.7 ± 0.5 ^{d,A}	91.9 ± 0.7 ^{d,A}	92.4 ± 0.3 ^{d,A}	92.7 ± 0.2 ^{d,A}
7	72.6 ± 0.5 ^{c,A}	71.2 ± 0.2 ^{c,A}	72.6 ± 0.4 ^{c,A}	75.4 ± 1.0 ^{c,B}	75.0 ± 0.9 ^{c,B}
14	69.3 ± 0.1 ^{c,B}	67.8 ± 0.8 ^{b,A}	69.6 ± 0.4 ^{b,B}	69.0 ± 0.4 ^{b,A,B}	68.5 ± 0.5 ^{b,A,B}
21	61.2 ± 2.3 ^{a,A}	65.8 ± 0.2 ^{a,B}	65.3 ± 0.2 ^{a,B}	63.7 ± 1.2 ^{a,b,A,B}	65.7 ± 0.3 ^{a,b,B}
28	65.0 ± 1.8 ^{b,A}	67.1 ± 0.7 ^{a,b,A}	66.0 ± 0.7 ^{a,A}	63.4 ± 4.2 ^{a,A}	64.9 ± 0.2 ^{a,A}
			a*RIND		
1	-4.2 ± 0.2 ^{a,A,B}	-4.5 ± 0.1 ^{a,A,B}	-4.2 ± 0.4 ^{a,A,B}	-4.6 ± 0.2 ^{a,B}	-3.9 ± 0.5 ^{a,A}
7	-6.3 ± 0.3 ^{d,A}	-5.8 ± 0.2 ^{b,c,A}	-6.3 ± 0.1 ^{c,A}	-6.1 ± 1.0 ^{c,A}	-6.0 ± 0.3 ^{c,A}
14	-5.4 ± 0.2 ^{c,A}	-5.9 ± 0.4 ^{c,A}	-5.7 ± 0.4 ^{b,c,A}	-5.6 ± 0.3 ^{b,c,A}	-5.9 ± 0.1 ^{c,A}
21	-4.8 ± 0.1 ^{b,A,B}	-5.7 ± 0.4 ^{b,c,C}	-5.4 ± 0.2 ^{b,B,C}	-4.8 ± 0.2 ^{a,A}	-5.1 ± 0.2 ^{b,A,B,C}
28	-5.2 ± 0.2 ^{b,c,A}	-5.1 ± 0.3 ^{a,b,A}	-5.3 ± 0.2 ^{b,A}	-5.0 ± 0.3 ^{a,b,A}	-5.2 ± 0.2 ^{b,A}
			b*RIND		
1	15.1 ± 0.3 ^{a,A}	15.4 ± 0.5 ^{a,A}	15.2 ± 0.7 ^{a,A}	16.0 ± 0.1 ^{a,A}	14.8 ± 0.6 ^{a,A}
7	21.9 ± 1.0 ^{c,A,B}	22.3 ± 0.2 ^{c,B}	21.5 ± 0.7 ^{b,A,B}	20.1 ± 0.9 ^{b,A}	21.0 ± 0.8 ^{c,d,A,B}
14	20.5 ± 0.3 ^{c,A}	22.0 ± 0.4 ^{c,B}	21.8 ± 0.7 ^{b,B}	21.8 ± 0.2 ^{b,B}	21.9 ± 0.6 ^{d,B}
21	18.0 ± 0.4 ^{b,A,B}	19.7 ± 0.2 ^{b,A,B}	15.2 ± 3.7 ^{a,A}	19.8 ± 0.2 ^{b,B}	19.2 ± 0.2 ^{b,A,B}
28	18.4 ± 0.6 ^{b,A}	19.7 ± 0.4 ^{b,A}	20.7 ± 0.5 ^{b,A}	20.6 ± 2.1 ^{b,A}	19.5 ± 0.6 ^{b,c,A}
			L*PASTE		

1	92.3 ± 0.1 ^{c,A}	92.0 ± 0.2 ^{b,A}	91.7 ± 0.7 ^{b,A}	92.6 ± 0.4 ^{b,A}	92.6 ± 0.5 ^{b,A}
7	83.5 ± 2.7 ^{a,A}	87.8 ± 0.2 ^{a,b,B}	87.0 ± 1.0 ^{a,A,B}	87.0 ± 1.2 ^{a,A,B}	86.0 ± 1.2 ^{a,A,B}
14	87.7 ± 0.4 ^{b,A}	87.7 ± 0.6 ^{a,b,A}	87.5 ± 0.2 ^{a,A}	86.4 ± 1.1 ^{a,A}	85.1 ± 2.7 ^{a,A}
21	84.9 ± 1.1 ^{a,b,A}	85.2 ± 0.9 ^{a,A}	85.9 ± 0.7 ^{a,A}	84.7 ± 3.2 ^{a,A}	85.6 ± 1.7 ^{a,A}
28	83.8 ± 0.4 ^{a,A}	85.8 ± 4.1 ^{a,A}	84.9 ± 2.7 ^{a,A}	85.4 ± 1.2 ^{a,A}	86.3 ± 0.6 ^{a,A}
			a*PASTE		
1	-3.7 ± 0.1 ^{a,A,B}	-3.9 ± 0.1 ^{a,B,C}	-4.1 ± 0.3 ^{a,b,C}	-3.3 ± 0.1 ^{a,A}	-4.8 ± 0.2 ^{a,D}
7	-4.9 ± 0.2 ^{b,c,A}	-4.8 ± 0.3 ^{a,A}	-4.8 ± 0.2 ^{b,A}	-4.9 ± 0.2 ^{b,A}	-4.7 ± 0.3 ^{a,A}
14	-4.4 ± 0.3 ^{b,A}	-4.4 ± 0.1 ^{a,A}	-4.2 ± 0.4 ^{a,b,A}	-4.1 ± 0.5 ^{a,b,A}	-4.2 ± 0.5 ^{a,A}
21	-4.8 ± 0.3 ^{b,c,A}	-4.5 ± 0.4 ^{a,A}	-4.7 ± 0.4 ^{a,b,A}	-3.8 ± 0.5 ^{a,A}	-4.6 ± 0.3 ^{a,A}
28	-5.4 ± 0.3 ^{c,A}	-4.5 ± 1.2 ^{a,A}	-4.0 ± 0.2 ^{a,A}	-4.1 ± 0.3 ^{a,b,A}	-4.3 ± 0.1 ^{a,A}
			b*PASTE		
1	13.8 ± 0.1 ^{a,A}	14.6 ± 0.2 ^{a,A,B}	15.2 ± 0.9 ^{a,B,C}	13.3 ± 0.5 ^{a,A}	16.0 ± 0.3 ^{a,C}
7	18.9 ± 1.3 ^{c,A}	18.4 ± 0.6 ^{a,A}	18.3 ± 0.1 ^{b,A}	17.3 ± 0.3 ^{b,A}	18.1 ± 0.4 ^{b,A}
14	16.4 ± 0.3 ^{b,A}	16.7 ± 0.3 ^{a,A}	16.4 ± 0.5 ^{a,A}	16.1 ± 0.6 ^{b,A}	16.6 ± 1.0 ^{a,b,A}
21	17.5 ± 0.6 ^{b,c,A}	16.9 ± 3.2 ^{a,A}	18.9 ± 0.3 ^{b,A}	16.7 ± 1.2 ^{b,A}	17.9 ± 1.0 ^{b,A}
28	19.1 ± 0.5 ^{c,A}	17.0 ± 3.1 ^{a,A}	16.0 ± 0.3 ^{a,A}	15.7 ± 0.2 ^{b,A}	16.6 ± 0.2 ^{a,b,A}

C-Control; N-cheese with natamycin; WC-cheese with SCW coating; WCO-cheese with SCW coating with oregano essential oil; WCS-cheese with SCW coating with clary sage essential oil. Different superscript letters (a,b,c,d) indicate significant differences for the same product in different ripening days. Superscript capital letters (A,B,C) indicate significant differences between products at the same ripening day.

Table S3. Texture parameters of cheese samples. Average values and standard deviation (±).

			HARDNESS (N)		
Storage time (days)	C	N	WC	WCO	WCS
1	8.4 ± 0.1 ^{a,A,B}	8.7 ± 0.3 ^{a,A,B}	8.1 ± 0.5 ^{a,A}	9.6 ± 0.8 ^{a,B}	8.2 ± 0.7 ^{a,A}
14	22.2 ± 1.6 ^{b,A}	18.9 ± 0.82 ^{b,B}	19.5 ± 2.4 ^{b,A,B}	15.9 ± 0.5 ^{b,C}	21.2 ± 2.1 ^{b,A,B}
28	24.0 ± 1.1 ^{b,B,C}	26.1 ± 2.5^{c,C}	17.6 ± 2.2 ^{b,A}	19.1 ± 0.6 ^{c,A}	20.0 ± 1.3 ^{b,A,B}
			ADHESIVENESS (N.s)		
1	-14.1 ± 4.6 ^{a,A}	-12.5 ± 1.3 ^{a,A}	-13.6 ± 2.4 ^{a,A}	-14.0 ± 3.5 ^{a,A}	-13.0 ± 5.2 ^{a,A}
14	-22.4 ± 4.5 ^{a,B}	-9.3 ± 2.8 ^{a,A}	-16.8 ± 4.6 ^{a,A,B}	-12.3 ± 1.5 ^{a,A}	-16.4 ± 0.7 ^{a,A,B}
28	-24.4 ± 4.7 ^{a,A}	-28.2 ± 5.5 ^{b,A}	-26.5 ± 1.8 ^{b,A}	-26.7 ± 2.9 ^{b,A}	-24.3 ± 2.4 ^{a,A}
			CHEWINESS (N.mm)		
1	4.27 ± 0.41 ^{a,A}	3.64 ± 0.30 ^{a,A}	3.99 ± 0.26 ^{a,A}	4.36 ± 0.41 ^{a,A}	3.48 ± 0.84 ^{a,A}
14	6.39 ± 0.78 ^{b,B}	3.09 ± 0.89 ^{a,A}	5.68 ± 0.64 ^{a,b,B}	4.65 ± 0.24 ^{a,A,B}	5.16 ± 0.84 ^{a,b,B}
28	8.62 ± 0.32 ^{c,A,B}	9.35 ± 0.96 ^{b,B}	6.67 ± 1.38 ^{b,A,B}	6.06 ± 0.50 ^{b,A}	7.02 ± 1.47 ^{b,A,B}
			GUMMINESS (N)		
1	4.3 ± 0.5 ^{a,A}	3.7 ± 0.3 ^{a,A}	4.0 ± 0.2 ^{a,A}	4.4 ± 0.4 ^{a,A}	3.5 ± 0.9 ^{a,A}
14	6.8 ± 0.9 ^{b,B}	3.2 ± 0.9 ^{a,A}	5.9 ± 0.5 ^{a,b,B}	4.8 ± 0.3 ^{a,A,B}	5.5 ± 1.0 ^{a,b,B}
28	8.9 ± 0.3 ^{c,A,B}	9.9 ± 0.7 ^{b,B}	6.8 ± 1.4 ^{b,A}	6.3 ± 0.4 ^{b,A}	7.2 ± 1.6 ^{b,A,B}
			COHESIVENESS		
1	0.517 ± 0.1 ^{b,A}	0.417 ± 0.0 ^{a,A}	0.493 ± 0.0 ^{c,A}	0.467 ± 0.1 ^{b,A}	0.433± 0.1 ^{a,A}
14	0.303 ± 0.0 ^{a,A}	0.457 ± 0.1 ^{a,B}	0.303 ± 0.0 ^{a,A}	0.300 ± 0.0 ^{a,A}	0.255 ± 0.0 ^{a,A}
28	0.373 ± 0.0 ^{a,A}	0.380 ± 0.1 ^{a,A}	0.383 ± 0.0 ^{b,A}	0.327 ± 0.0 ^{a,A}	0.357 ± 0.1 ^{a,A}
			RESILIENCE		
1	0.067 ± 0.0 ^{b,B}	0.047 ± 0.0 ^{a,A}	0.053± 0.0 ^{b,A,B}	0.053 ± 0.0 ^{b,A,B}	0.043 ± 0.0 ^{a,A}
14	0.023 ± 0.0 ^{a,A}	0.037 ± 0.0 ^{a,b,B}	0.027 ± 0.0 ^{a,A,B}	0.030 ± 0.0 ^{a,A,B}	0.028 ± 0.0 ^{a,A,B}
28	0.027 ± 0.0 ^{a,A}	0.027 ± 0.0 ^{b,A}	0.027± 0.0 ^{a,A}	0.030 ± 0.0 ^{a,A}	0.027 ± 0.0 ^{a,A}

C-Control; N-cheese with natamycin; WC-cheese with SCW coating; WCO-cheese with SCW coating with oregano essential oil; WCS-cheese with SCW coating with clary sage essential oil. Different superscript letters (a,b,c) indicate significant differences for the same product in different ripening days. Superscript capital letters (A,B,C) indicate significant differences between products at the same ripening day.

Table S4. Microbiological counts of cheese samples (log CFU/g). Average values and standard deviation (±).

			LACTOCOCCI		
Storage time (days)	C	N	WC	WCO	WCS
1	8.5 ± 0.0 [a,A]	8.5 ± 0.0 [a,A]	9.7 ± 0.0 [b,B]	8.5 ± 0.3 [a,A]	9.1 ± 0.1 [b,c,A,B]
7	9.3 ± 0.1 [b,B]	9.1 ± 0.0 [b,A,B]	8.7 ± 0.3 [a,A]	8.6 ± 0.2 [a,b,A]	8.7 ± 0.1 [a,A]
14	9.3 ± 0.1 [b,A]	9.9 ± 0.1 [c,B,C]	9.7 ± 0.0 [b,B]	9.1 ± 0.0 [b,c,A]	10.0 ± 0.0 [d,C]
21	8.8 ± 0.1 [a,b,A,B]	8.7 ± 0.0 [a,b,A]	8.6 ± 0.0 [a,A]	9.5 ± 0.0 [c,C]	8.9 ± 0.0 [a,b,B]
28	8.6 ± 0.3 [a,A]	8.7 ± 0.2 [a,b,A,B]	9.3 ± 0.1 [b,B,C]	8.9 ± 0.1 [a,b,c,A,B,C]	9.4 ± 0.0 [c,C]
			LACTOBACILLI		
1	8.5 ± 0.0 [a,A,B]	7.6 ± 0.2 [a,A]	9.5 ± 0.0 [b,B]	7.8 ± 0.6 [a,A]	8.5 ± 0.7 [a,A,B]
7	9.5 ± 0.0 [b,B]	9.5 ± 0.0 [c,d,B]	9.2 ± 0.2 [a,b,B]	9.1 ± 0.1 [a,b,B]	8.6 ± 0.0 [a,A]
14	9.3 ± 0.0 [b,A,B]	9.9 ± 0.0 [d,B]	8.8 ± 0.1 [a,A]	9.5 ± 0.5 [b,A,B]	9.4 ± 0.2 [a,A,B]
21	8.5 ± 0.2 [a,A]	8.9 ± 0.0 [b,A,B]	8.8 ± 0.1 [a,A,B]	9.7 ± 0.1 [b,C]	9.0 ± 0.1 [a,B]
28	8.8 ± 0.0 [a,A]	9.0 ± 0.3 [b,c,A]	9.3 ± 0.0 [b,A]	9.2 ± 0.0 [b,A]	9.2 ± 0.0 [a,A]
			TOTAL MESOPHILIC AEROBIC COUNTS		
1	8.0 ± 0.0 [b,A,B]	8.0 ± 0.1 [a,A,B]	9.0 ± 0.0 [c,C]	7.8 ± 0.4 [b,A]	8.5 ± 0.0 [a,B,C]
7	8.1 ± 0.3 [b,A]	8.2 ± 0.4 [a,A]	7.8 ± 0.3 [b,A]	7.9 ± 0.6 [b,A]	7.7 ± 0.5 [a,A]
14	8.3 ± 0.2 [b,C]	7.8 ± 1.2 [a,B,C]	6.3 ± 0.3 [a,A]	6.7 ± 0.3 [a,A,B]	7.7 ± 0.7 [a,A,B,C]
21	8.0 ± 0.2 [b,A]	8.1 ± 0.4 [a,A]	8.0 ± 0.5 [b,A]	8.0 ± 0.7 [b,A]	8.4 ± 0.3 [a,A]
28	7.3 ± 0.4 [a,A]	7.6 ± 0.7 [a,A]	8.0 ± 0.6 [b,A]	7.8 ± 0.2 [b,A]	8.0 ± 0.2 [a,A]
			YEASTS AND MOULDS		
1	3.7 ± 1.2 [a,A]	3.5 ± 0.7 [a,A]	3.0 ± 0.1 [a,A]	3.2 ± 0.2 [a,A]	4.0 ± 1.4 [a,A]
7	4.5 ± 0.3 [a,b,A]	4.2 ± 0.5 [a,b,A]	4.5 ± 0.7 [b,A]	4.6 ± 0.3 [b,A]	4.7 ± 0.3 [a,b,A]
14	6.3 ± 0.2 [c,B]	4.9 ± 0.8 [b,c,A]	5.5 ± 0.6 [b,A,B]	5.0 ± 0.4 [b,A]	5.4 ± 0.1 [b,c,A,B]
21	5.8 ± 0.9 [b,c,A]	5.4 ± 0.6 [b,c,A]	5.4 ± 0.6 [b,A]	5.4 ± 0.2 [b,c,A]	6.0 ± 0.2 [b,c,A]
28	6.0 ± 0.3 [c,A]	5.6 ± 0.4 [c,A]	6.7 ± 0.5 [c,A]	6.3 ± 0.9 [c,A]	6.2 ± 0.4 [c,A]

C-Control; N-cheese with natamycin; WC-cheese with SCW coating; WCO-cheese with SCW coating with oregano essential oil; WCS-cheese with SCW coating with clary sage essential oil. Different superscript letters (a,b,c) indicate significant differences for the same product in different ripening days. Superscript capital letters (A,B,C) indicate significant differences between products at the same ripening day.
